# Physician migration at its roots: a study on the factors contributing towards a career choice abroad among students at a medical school in Pakistan

**DOI:** 10.1186/1744-8603-8-43

**Published:** 2012-12-15

**Authors:** Asfandyar Sheikh, Syed Hassan Abbas Naqvi, Kainat Sheikh, Syed Hassan Shiraz Naqvi, Muhammad Yasin Bandukda

**Affiliations:** 1 Dow Medical College, Dow University of Health Sciences, Baba-e-Urdu Road, Karachi, Pakistan; 2 Sindh Medical College, Dow University of Health Sciences, Rafiqui H.J. Shaheed Road, Karachi, Pakistan

## Abstract

**Background:**

Physician migration, also known as “brain drain,” results from a combination of a gap in the supply and demand in developed countries and a lack of job satisfaction in developing countries. Many push and pull factors are responsible for this effect, with media and internet playing their parts. Large-scale physician migration can pose problems for both the donor and the recipient countries, with a resulting reinforcement in the economic divide between developed and developing countries. The main objectives of our study were to determine the prevalence of migration intentions in medical undergraduates, to elucidate the factors responsible and to analyze the attitudes and practices related to these intentions.

**Methods:**

This was a cross-sectional, observational, questionnaire-based study, conducted at Dow Medical College of Dow University of Health Sciences, Karachi, between January, 2012 and May, 2012. A total of 323 students responded completely. The questionnaire consisted of 3 sections, and was aimed at collecting demographic details, determining students’ migratory intentions, evaluating reasons for and against migration and assessing attitudes and practices of students related to these intentions.

**Results:**

Out of 323 respondents, 195 wanted to pursue their careers abroad, giving a prevalence rate of 60.4% in our sample. United States was the most frequently reported recipient country. The most common reasons given by students who wished to migrate, in descending order, were: lucrative salary abroad followed by quality of training, job satisfaction, better way of life, relatives, more opportunities, better working environment, terrorism in Pakistan, harassment of doctors in Pakistan, desire to settle abroad, more competition in Pakistan, better management, peer pressure, longer working hours in Pakistan, religious reasons, parent pressure, political reasons and favoritism in Pakistan. A considerable number of respondents had already started studying for licensing examinations, and were also planning of gaining clinical experience in their desired country of interest.

**Conclusion:**

Physician migration is a serious condition that requires timely intervention from the concerned authorities. If considerable measures are not taken, serious consequences may follow, which may pose a threat to the healthcare system of the country.

## Introduction and background

Medicine is one of the noblest professions man can adopt. Doctor’s undergo a rigorous period of undergraduate training, which determines how successful they will be in their careers. The training period varies from country to country, ranging from 5 years in Pakistan to 7-8 years in the United States (including pre-medical years) [1,2]. However, a doctor’s life is never at ease, as after this intensive period of training, comes an even more strenuous professional phase, requiring as much as 80 hours per week (during residency) [3]. Under these circumstances, it is only natural for doctors to have a higher level of expectations than most other people, most of which revolve around a better salary, better recognition and a better way of life.

Physician migration, also known as the “brain drain,” is not a new phenomenon. However, it has only recently been highlighted as a problem for both the donor and the recipient countries, due to a larger number of doctors migrating or intending to migrate compared to the past [4]. This increase has been substantiated by the expansion of the gap between demand and supply of physicians in developed countries. This, coupled with the lack of job satisfaction in developing countries, stimulates a wide-scale exodus of physicians leading to a further economic divide between developed and developing countries [5].

**Figure 1:**
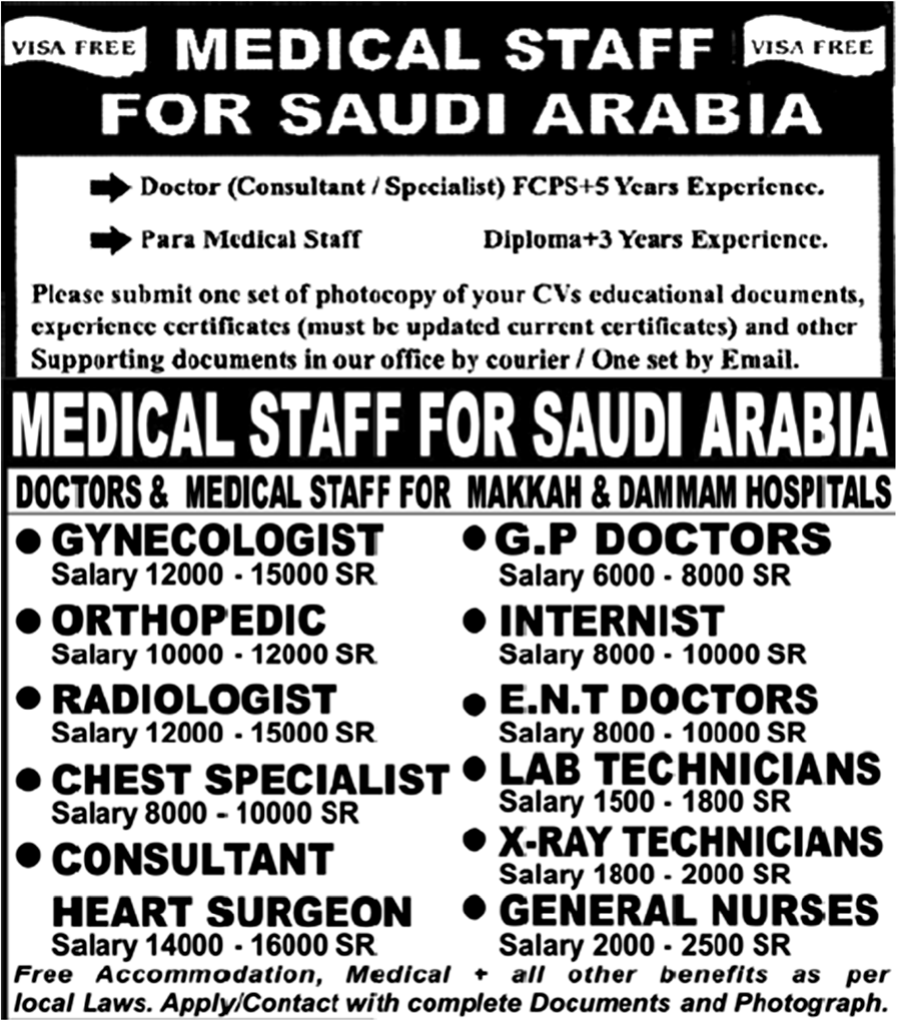
An advertisement in a local newspaper for positions abroad.

Certain “push” and “pull” factors from donor and recipient countries are responsible for this effect [6]. For example, poor salary structure and poor quality of training were the most common push factors reported by Syed and colleagues in a study conducted at two private medical colleges in Karachi [7]. Similarly, a stronger history of violent events and harassment was found in doctors opting to migrate in a study conducted in Iraq [8]. In addition to these, certain pull factors of the recipient countries such as favorable immigration policies, better way of life and better quality of training serve as an icing on the cake [9-12]. For example, better job opportunities were the second most frequently reported reason for migration in a study conducted in South Africa [13]. Under these circumstances, it is appropriate to state that apart from the push and pull factors mentioned above, print and electronic media may also play an important part in facilitating migration intentions, although their role is yet to be appreciated scientifically. For example, in the absence of proper Government regulations and limitations, foreign companies infinitely advertise open positions in local newspapers (see Figure 1), often promising lucrative salaries and benefits, which serve the role of adding fuel to fire. Furthermore, the recent “boom” of television channels broadcasting shows depicting a better quality of life at foreign destinations has not only led to an escalation of migration intentions, but has also led to an increased sense of dissatisfaction with the current setup.

Skilled Migration has an everlasting impact on both the donor and the recipient. The donors are often developing countries, who spend a fortune on educating future doctors. For example, the Government of Pakistan spends a hefty amount on subsidizing undergraduate medical education. Not only does physician migration cause serious damages to a country’s economy, it also leads to the crumbling of its healthcare system by taking away its most fundamental units. The number of doctors is often more than what the recipient can support, which leads to them adopting other (often less dignified) professions, with some becoming illegal immigrants [14].

Under these circumstances, we feel that it is worthwhile to introduce the healthcare system of Pakistan before proceeding with the discussion. The controversial WHO report of 2000 placed Pakistan at number 122 in the rankings of healthcare systems, ahead of countries like Russia and China [15]. However the overall condition of the system still attracts a great deal of criticism from all circles. The Pakistan Medical and Dental Council (PMDC) is the body responsible for registering medical practitioners and for maintaining the highest degree of medical practice. The private sector accounts for the majority of healthcare needs, catering to approximately 80% of all outpatient visits [16]. The provincial health departments are responsible for planning and fund allocation in the public sector, which were, up till 2011, controlled by the Ministry of Health (MOH) [16]. The health expenditure in 2007-08, according to the MOH, amounted to Rs3.791 billion whereas the development expenditure amounted to Rs14.272 billion [17]. However, the available health and sanitation facilities in the rural and urban areas are still far from adequate which is reflected in the high infant mortality (76.7/1,000 persons) and maternal mortality (350/100,000 live births) rates for the country [17].

Reports suggest that as many as 15,813 physicians from Pakistan were practicing abroad in 2005, constituting 17.6% of the total pool of physicians [18]. United States is the most preferred recipient country by International Medical Graduates (IMGs). According to an estimate, about one fourth of the physician workforce in USA comprises of IMGs [19]. India, Philippines and Pakistan are among the topmost sources. Pakistanis comprise the fourth highest source of IMG doctors in the US, constituting 4.8% of the total pool [20]. Internal Medicine is the most common specialty chosen, with IMGs filling in 37.7% of categorical positions [21]. These statistics often lead to a bias in career choice of undergraduates wishing to migrate, forming doctors who lack motivation as a result of adopting a career that they originally did not intend to [22].

### Rationale and objectives

Previous studies have reported a high prevalence of migration intention among medical college students [7,23-25]. Since college life marks the beginning or “roots” of a medical career, a high prevalence rate at this level is no less than a warning sign. However, it is worthwhile to note here that proper interventions at this level may prove to be extremely rewarding, as the old saying goes “nip the evil in the bud.”

Certain limitations with previous studies forced the authors to design this study. For example, the study of Syed et al. was conducted at private institutions, and in doing so it overlooks the impact of tuition fees on migration intentions [7]. Similarly, Syed et al. and Imran et al. failed to include earlier years in their study population [7,23]. Furthermore, none of the studies previously conducted place the required emphasis on the practices of students intending migration. With the above in mind, the investigators sought:

• to determine the prevalence of migration intentions in medical undergraduates,

• to elucidate the factors responsible for these intentions and

• to analyze the attitudes and practices related to these intentions.

## Materials and methods

### Study design and setting

This was a cross sectional, questionnaire-based study conducted from 23^rd^ January, 2012 to 2^nd^ May, 2012 at Dow Medical College, which is part of the government-run Dow University of Health Sciences, Karachi. This college has been known for producing quality professionals, who practice in Pakistan and abroad. The MBBS program of Dow Medical College spans over a period of 5 years, with an additional year of internship. The college accepts admissions in three categories: on merit (based on an admission test and previous academic performance), on self-finance and on foreigner’s seat. Those on merit pay Rs20,000 (~USD200) per annum as tuition fee, while those on self-finance and those on foreigners’ seats pay Rs280,000 (~USD3,000) and Rs1,250,000 (~USD13,000) respectively.

### Operational definitions

#### Roots

The Oxford Dictionary defines “root” as “the basic cause, source, or origin of something” [26]. In our context, the word “roots” implies early educational levels (or origins) of medicine.

#### International medical graduate

An IMG is a graduate from a medical school outside the United States ie from a a school that is not accredited by the Liaison Committee on Medical Education [27].

#### Push factors

Factors in a health system or country that repel or facilitate the movement of health workers away from that system or country.

#### Pull factors

Factors in a health system or country that attract or facilitate the movement of health workers towards that system or country.

#### Licensing examinations

Examinations that must be passed in order to practice medicine in a country.

### Study tool

The study tool was designed with the help of the Department of Community Medicine, Dow University of Health Sciences. Extensive keyword search was undertaken on Pubmed and Google Scholar in order to draft the initial questionnaire. The keywords utilized were “reasons for physician migration”, “brain drain”, “health personnel migration”, “migration intentions among students.” The factors that were responsible for staying or migration were also ascertained by a thorough search of previous literature on the topic [28-31]. Studies conducted by Syed, Imran and Akl served as templates for push and pull factors [7,23,24].

The questionnaire was divided into 3 sections. Section 1 was concerned with the demographic data of the participants, which consisted of age, sex, study year and whether they possessed a foreign nationality or not. Section 2A and 2B formed the skeleton of the questionnaire. Section 2A dealt with the reasons for staying in Pakistan while Section 2B dealt with the reasons for pursuing a career abroad. A list of 18 push or pull factors were included in a tabulated form for both the subsections and each was scored on a Likert scale of 1-5, with 1 denoting strong disagreement for a reason, 2 denoting a lesser degree of disagreement, 3 denoting neutrality, 4 denoting a lesser degree of agreement and 5 expressing strong affirmation for a reason. These tables were followed by a list of yes-no questions for each subsection, that were aimed at evaluating the extent to which each participant wanted to stay in Pakistan or migrate. Two “emotive” questions were also added, each with three choices of answers (“yes”, “no” and “it feels bad the way you put it, but the answer is still yes”). The last section assessed the attitudes, practices and expectations of the students regarding the preparation for the exams and electives in their desired country of interest.

An undeclared pretest of the questionnaire was conducted on a sample of 15 students in the waiting area. Open ended questions were included in order to fully extract the opinion of the respondents. The questionnaire was revised accordingly in order to ensure the best possible form.

The final questionnaire demonstrated intermediate internal consistency. Cronbach’s alpha was calculated for the final data, which came out to be 0.691 for Section 2A and 0.649 for Section 2B.

#### Scoring

Final score was calculated by adding the Likert scores of all the perceived reasons in the tables for a particular student.

### Study population and participants

The intended target population of our study consisted of all students enrolled in medical colleges in Pakistan along with interns practicing in the associated hospitals. However, due to logistic reasons, the study population was limited to all students belonging to 1^st^ through 5^th^ (final) years in Dow Medical College. Interns from the Civil Hospital, Karachi (the teaching Hospital associated with Dow Medical College) were also included.

#### Inclusion and exclusion criteria

All those students, who were willing to participate in the study and gave their consent, were included. However those students, who were undergoing physical and/or mental stress (for example due to failure in an exam, or due to death of a relative), were excluded. All interns who had not graduated from Dow Medical College or who had been studying in the college for less than 6 months were also excluded.

### Ethical review

The Ethical Review Board of Dow University of Health Sciences approved the study.

### Study protocol

The sample size was calculated by taking 95% confidence level and 5% margin of error, for a population size of 1,600 (300 for each year and 100 for interns), which came out to be 310. The number was rounded off to 400 to account for an expected response rate of approximately 75%.

**Figure 2:**
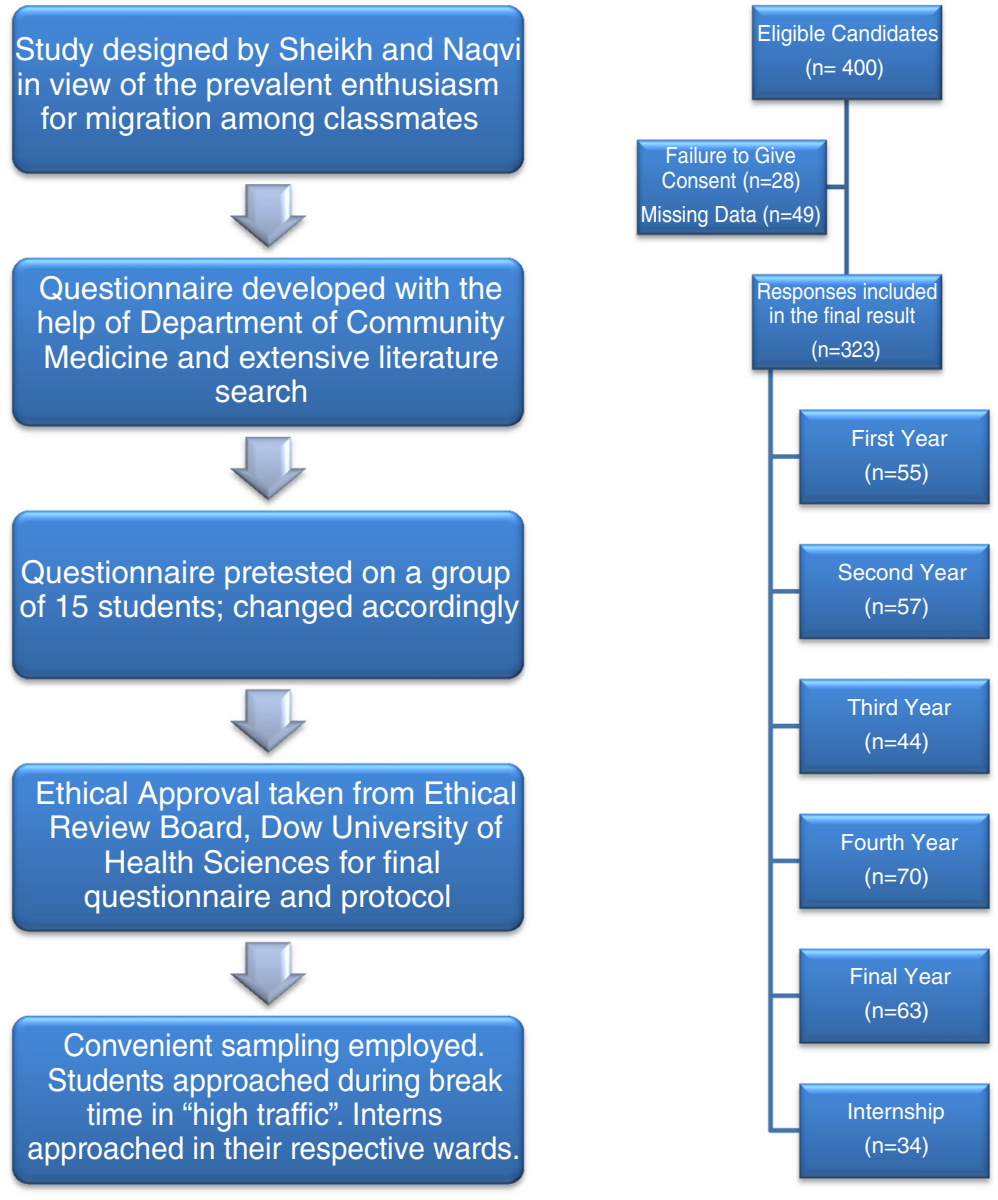
Logical sequence of methodology.

Since the investigators did not have access to the registration data (including enrolment numbers, which could be used to generate a random sample) of the students, and the demographics of the respondents were elicited only after interview, randomization was not performed. Those in close proximity to the interviewers were included. Thus, convenient sampling was employed for the study. Students were approached during break time and were administered the questionnaire in “high traffic” areas such as playgrounds, food-courts, library, digital arena, reading halls, waiting area and parking. Interns were approached in their respective wards. All those who were willing to participate were fully explained the objectives, protocol and consequences of the study and were assured about the anonymity and confidentiality of their answers. Informed, written consent was obtained from all participants. Figure 2 gives a graphical representation of the logical sequence of “Methods”.

### Analysis of data

Despite special emphasis being placed at the time of administration on the completion of the questionnaire in entirety, some of the questionnaires did have missing fields, and were duly excluded. Data from the questionnaire was entered in SPSS (Statistical Package for the Social Sciences) version 14 for analysis. Descriptive statistics (means, standard deviations and percentages) formed the mainstay of the statistical analysis.

## Results

### Demographics

A total of 323 questionnaires were returned (response rate 80.8%). Mean age was 21.2 ± 2.3 years. Majority (53.9%) consisted of females. 17.0% (55) of the respondents were from first year, 17.6% (57) from second year, 13.6% (44) from third year, 21.7% (70) from fourth year, 19.5% (63) from final year and 10.5% (34) were doing internship. 164 (50.7%) respondents were on merit, 121 (37.5%) on self-finance and 38 (11.8%) were foreign nationals. 41 respondents were married.

Out of 323 respondents, 195 wanted to pursue their careers abroad, giving a prevalence rate of 60.4% in our sample. Table 1 provides a summary of migratory intentions of the respondents.

**Table 1:** Migratory intentions of students

		**Career Abroad?**				
		**Yes**		**No**		**Total**
		**Male**	**Female**	**Male**	**Female**	
		**N(%)**	**N(%)**	**N(%)**	**N(%)**	**N(%)**
Year	1	13(4.0)	24(7.4)	12(3.7)	6(1.9)	55(17.0)
	2	17(5.3)	8(2.5)	12(3.7)	20(6.2)	57(17.6)
	3	5(1.5)	19(5.9)	15(4.6)	5(1.5)	44(13.6)
	4	19(5.9)	24(7.4)	10(3.1)	17(5.3)	70(21.7)
	5	19(5.9)	27(8.4)	7(2.2)	10(3.1)	63(19.5)
	Internship	5(1.5)	15(4.6)	7(2.2)	7(2.2)	34(10.5)
Nationality	Pakistani	64(19.8)	97(30.0)	63(19.5)	61(18.9)	285(88.2)
	Foreign	14(4.3)	20(6.2)	0	4(1.2)	38(11.8)
Merit	Yes	49(15.2)	60(18.6)	26(8.0)	29(9.0)	164(50.8)
	No	29(9.0)	57(17.6)	37(11.5)	36(11.1)	159(49.2)
Total		78(24.1)	117(36.2)	63(19.5)	65(20.1)	323(100)

### Career choice

Table 2 provides a summary of future career choices of the respondents in tabulated form. Surgery (43.3%) was the most common field, followed by Internal Medicine (30.7%), Pediatrics (8.0%), Gynecology and Obstetrics (4.3%), Psychiatry (3.4%), Anesthesiology (3.1%), Radiology (1.9%), Otolaryngology (1.5%), Ophthalmology (0.9%) and Orthopedics (0.6%). More than half (51.8%) of the students interviewed reported that their career choice was flexible and could change in the future.

**Table 2:** Career choices of students

	**Year**												
	**1**		**2**		**3**		**4**		**5**		**Internship**		**Total**
	**Male**	**Female**	**Male**	**Female**	**Male**	**Female**	**Male**	**Female**	**Male**	**Female**	**Male**	**Female**	
	**N(%)**	**N(%)**	**N(%)**	**N(%)**	**N(%)**	**N(%)**	**N(%)**	**N(%)**	**N(%)**	**N(%)**	**N(%)**	**N(%)**	**N(%)**
Internal Medicine	9(2.8)	2(0.6)	7(2.2)	5(1.5)	3(0.9)	5(1.5)	13(4.0)	10(3.1)	11(3.4)	12(3.7)	5(1.5)	17(5.3)	99(30.7)
Surgery	8(2.5)	8(2.5)	25(7.7)	20(6.2)	14(4.3)	6(1.9)	13(4.0)	12(3.7)	10(3.1)	19(5.9)	0	5(1.5)	140(43.3)
Pediatrics	1(0.3)	1(0.3)	0	0	0	0	0	6(1.9)	5(1.5)	6(1.9)	7(2.2)	0	26(8.0)
Dermatology	0	3(0.9)	0	0	0	0	0	0	0	0	0	0	3(0.9)
Gynae and Obs	0	6(1.9)	0	0	0	0	0	8(2.5)	0	0	0	0	14(4.3)
Orthopedics	2(0.6)	0	0	0	0	0	0	0	0	0	0	0	2(0.6)
Radiology	0	2(0.6)	0	0	0	4(1.2)	0	0	0	0	0	0	6(1.9)
Psychiatry	0	7(2.2)	0	0	0	4(1.2)	0	0	0	0	0	0	11(3.4)
Anesthesiology	2(0.6)	0	0	0	3(0.9)	5(1.5)	0	0	0	0	0	0	10(3.1)
Ophthalmology	3(0.9)	0	0	0	0	0	0	0	0	0	0	0	3(0.9)
ENT	0	0	0	0	0	0	0	5(1.5)	0	0	0	0	5(1.5)
Other	0	1(0.3)	0	0	0	0	3(0.9)	0	0	0	0	0	4(1.2)
Total	25(7.7)	30(9.3)	32(9.9)	25(7.7)	20(6.2)	24(7.4)	29(9.0)	41(12.7)	26(8.0)	37(11.5)	12(3.7)	22(6.8)	323(100)

### Career in Pakistan

Table 3 provides a summary of potential risk factors for pursuing career in Pakistan, along with their mean scores.

**Table 3:** Reasons for staying

	**Mean**	**S.D.**
Relatives	3.84	1.350
Wealth and Properties	1.62	0.981
Loan to Settle	1.03	0.354
Peer Pressure	1.51	0.896
Lack of Resources	3.69	1.484
Visa Problems	1.23	0.715
Better Way of Life	2.57	1.581
Racism Abroad	2.93	1.698
Lack of Opportunities Abroad	1.72	0.996
No Top Positions for IMGs Abroad	2.28	1.229
Long Working Hours Abroad	3.15	1.700
Desire to Serve Country	2.38	1.420
Political Reasons	1.61	0.916
Religious Reasons	1.90	1.128
Parent Pressure	1.87	1.082
More Competition Abroad	1.70	0.873
More Job Satisfaction	3.26	1.628
Favoritism Abroad	1.91	1.239
Other	1.39	0.872
Total Score	41.58	12.879

### Pull Factors

The most common pull factor reported by the interviewees as having at least some impact on their decision was strong family ties (65.6% of those who wished to stay) followed by lack of resources (61.7%), job satisfaction (50.0%), better way of life (31.3%), desire to serve country (28.9%), religious reasons (10.9%), parent pressure (7.0%), wealth in Pakistan (3.9%), peer pressure (2.3%) and loans to settle (0.8%).

### Push Factors

The most common push factor reported by the interviewees as having at least some impact on their decision was long working hours abroad (45.3% of those who wished to stay) followed by racism abroad (44.5%), no top positions for IMGs abroad (25.8%), favoritism abroad (14.1%), lack of opportunities abroad (5.5%), more competition abroad (4.7%), political reasons (3.1%) and visa problems (1.6%).

Only 31 (24.2%) interviewees believed that the above mentioned reasons could be overcome in the future. 51.6% (66) believed that their decision to stay was flexible.

### Attitudes of the respondents

A large proportion of the respondents [94 (73.4% of those who did not wish to migrate)] reported that they would leave Pakistan if they were disheartened by policies in effect. 109 (85.2%) respondents had at least one relative abroad. The most commonly cited reason for not migrating, despite having relatives abroad was relatives not being close enough (43.8%), followed by none of the choices mentioned (30.5%), other reasons (21.1%) and relatives not being well-settled themselves (4.7%). 43 respondents would advise others to stay in Pakistan as well.

When asked, “Are you willing to stay in Pakistan even after knowing that you may earn only a fraction of what you might earn abroad?” most interviewees (57.0%) responded with a “yes”. However, when asked if they were willing to stay after knowing that they might be a target of terrorism, most (63.2%) responded with “it feels bad the way you put it, but the answer is still yes”.

### Career abroad

**Figure 3:**
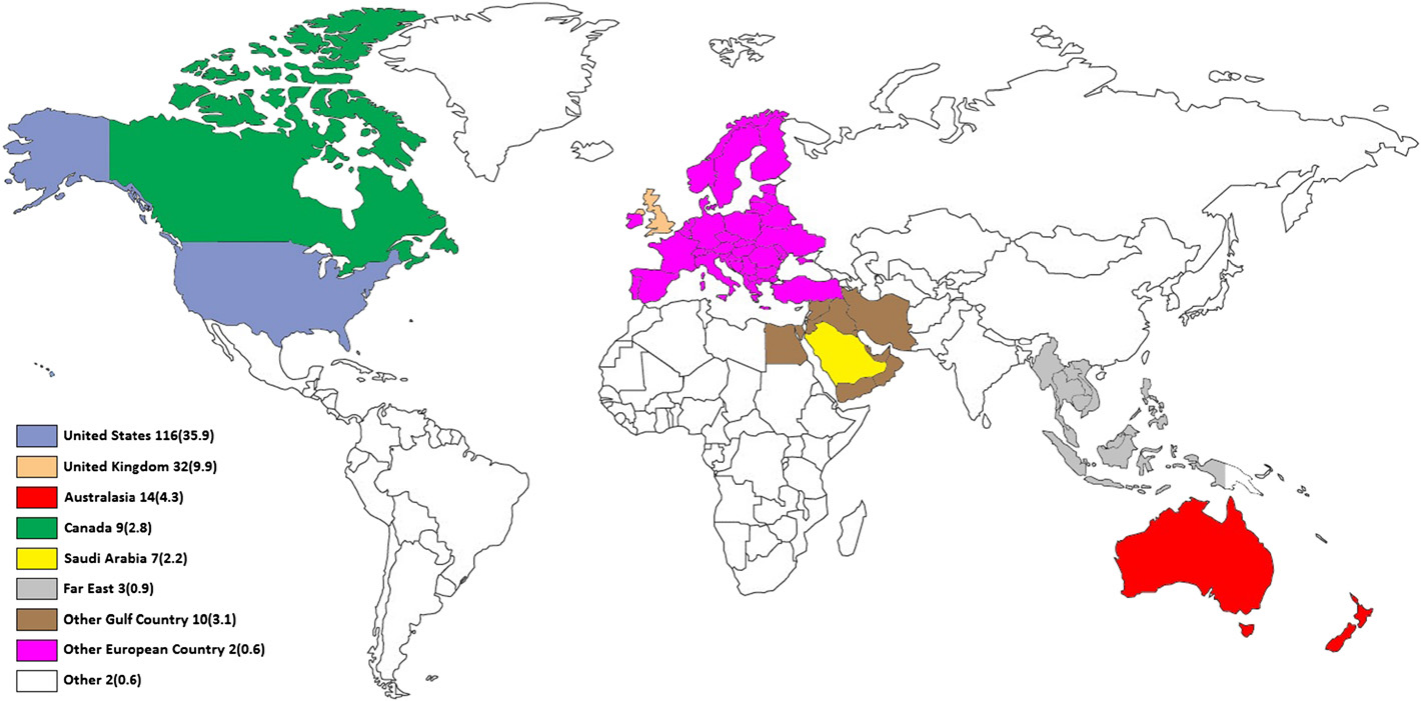
Percentages of favorite target countries.

The most favorite primary target country was United States (35.9% of total). Figure 3 gives a graphical representation of the percentages of favorite target countries. Table 4 provides a summary of risk factors for pursuing career abroad, with their mean scores.

**Table 4:** Reasons for migration

	**Mean**	**S.D.**
Relatives	3.23	1.706
Lucrative Salary	4.23	1.031
Harassment of Doctors in Pakistan	3.01	1.489
Peer Pressure	2.12	1.048
Quality of Training	3.89	1.365
Better Working Environment	2.97	1.674
Better Way of Life	3.48	1.524
Terrorism in Pakistan	3.07	1.518
More Opportunities	3.31	1.410
Better Management	2.19	1.358
Long Working Hours in Pakistan	1.88	1.031
Desire to Settle Abroad	2.84	1.459
Political Reasons	1.23	0.698
Religious Reasons	1.46	0.915
Parent Pressure	1.35	0.832
More Competition in Pakistan	2.61	1.433
More Job Satisfaction	3.56	1.560
Favoritism in Pakistan	1.60	0.808
Other	1.13	0.486
Total Score	49.15	14.554

### Pull factors

The most common pull factor reported by the interviewees as having any degree of impact on their decision was lucrative salary abroad (76.4% of those who wished to migrate) followed by quality of training (69.2%), job satisfaction (59.5%), better way of life (55.4%), relatives abroad (50.3%), more opportunities (49.2%), better working environment (48.2%), desire to settle abroad (36.4%), better management (31.3%) and religious reasons (4.6%).

### Push factors

The most common push factor reported by the interviewees as having any degree of impact on their decision was terrorism in Pakistan (40.0% of those who wished to migrate) followed by harassment of doctors in Pakistan (39.0%), more competition in Pakistan (35.9%), peer pressure (9.7%), longer working hours in Pakistan (5.1%), parent pressure (4.1%), political reasons (3.1%) and favoritism in Pakistan (2.1%).

Only 74 (37.9%) interviewees believed that the above mentioned reasons could be overcome in the future. 43.1% (84) believed that their decision to migrate was flexible.

### Attitudes of the respondents

A considerable proportion of the respondents [72 (36.9% of those who wished to migrate)] reported that their career choice was biased due to competition in other specialties abroad. 17 (8.7%) respondents answered that they were ready to give up medicine for a less dignified profession (e.g. taxi driver) if need be. A striking 57.9% of the interviewees reported that they would make more than one attempt to migrate (if their previous attempts failed). 131 students said that they would apply for the nationality of their country of choice, with 46 even willing to relinquish their Pakistani citizenship if need be. 107 said that they would also call their family after acquiring citizenship. 94 said that they would return to Pakistan after a certain amount of time, while 113 said that they would advise others to leave Pakistan as well.

When asked, “Are you willing to migrate even after knowing that Pakistan already has a low doctor-patient ratio?” most interviewees (71.2%) opted for “it feels bad the way you put it, but the answer is still yes”. However, when asked if they were willing to migrate after knowing that they might be a target of racism, most (56.7%) responded with a “yes”.

### Preparatory measures, attitudes and practices of respondents related to migratory intentions

USMLE (68.2% of those who wished to migrate) was the most common primary examination that the respondents aimed to give, followed by PLAB (14.9%), MCCEE (4.6%) and AMC (3.6%). About 36.4% (71) wanted to appear for the above mentioned exams before graduation. The reported mean number of years that the respondents aimed to appear for the first part of these examinations was 2.74 ± 1.890 years. 53.8% (105) had already started preparing for these examinations. Out of these, 49.5% (52) were using a special course for the preparation, 21.9% (23) were studying in groups and 58.1% (61) gave less importance to the University Exams. The mean number of hours spent on preparing for the above mentioned examinations was 3.95 ± 2.159. 21 (10.8% of those who wished to migrate) had already performed at least one elective/externship/observership in their country of choice. A staggering 83.6% (163) wished to perform electives in the future. The mean number of years in which they aimed at performing these electives was 1.39 ± 1.198 years. The mean amount of money that the respondents were ready to spend for these exams/electives was Rs339,538 ± 292,846.486 (~USD3,500 ± 3000).

About 65.6% of those who wished to migrate had received some form of guidance regarding the above mentioned exams/electives. Websites were the most common medium of this guidance, followed by seniors and seminars. 56.9% (111) reported that they were under any degree of stress due to these exams/electives. 42.1% (82) were even ready to skip their internships in order to prepare for them. 78.5% (153) said that they would make more than one attempt at passing the above mentioned exams (if their previous attempts failed).

## Discussion

This is the first study of its kind in our country, that aims to provide an in depth analysis of physician migration, with a particular emphasis on the attitudes and practices undertaken by the students in pursuit of their wishes. The prevalence rate of migratory intentions in our sample was 60.4%, which was similar to that of King Edward Medical College, and Baqai Medical College, but different from that of Aga Khan University [7,23]. It was also consistent with data from other countries such as India and South Africa [25,32]. Females formed the majority of our sample, which corresponds to the higher number of females who get admission at the college. The most common specialty preferred by the students was Surgery. This data is consistent with a previous study conducted in 2011, showing that the momentum has not shifted after 1 year [33]. The top destination choice among those who wished to migrate was the United States [34]. This finding is similar to that of Rao for students from India [25]. The reason behind this is the fact that the United States has some of the most favorable policies for temporary as well as permanent skilled migrants [35].

Among reasons for pursuing a career abroad, the mean score for “lucrative salary” was the highest. It was higher in males (4.69) compared to females (3.83), and in married (4.85) compared to unmarried (4.14). It should be noted here that medical graduates in Pakistan earn a meager Rs24,000/month (~USD250) during their house-job and Rs42,500/month (~USD500) during post-graduation [36]. Recently, strikes erupted throughout the country over the issue of increasing the salary of doctors [37]. Thus, it seems that as a last resort, doctors are forced to migrate, in search of a better livelihood and to support themselves and their families. Financial concerns were also cited as an important reason for facilitating migration in studies conducted by Jośko et al. on Polish students and by Labugo et al. on Ugandan physicians [38,39].

Other reasons for migration included a better quality of training and a better way of life abroad. Although the situation in Pakistan is a lot better than it was in the early 70s, the quality of post-graduation training provided can under no circumstances be deemed satisfactory. As a result, physicians, no matter how patriotic, are forced to migrate in order to quench their everlasting thirst for knowledge. Moreover, the recent “boom” of TV channels and other forms of media has brought about a wave of change in the mentality of the masses, with more people now desiring a better way of life. This wave has affected the physicians as well, who believe that there should be some sort of remuneration in the form of a better quality of life, for all the hours they put into work.

Previous studies conducted on professional physicians have yielded similar results. For example, in a study conducted on Iranian physicians practicing in the United States, Ronaghy et al. found out that a lack of professional working environment was one of the most common reasons for not returning [40]. In contrast, a study conducted on foreign physicians practicing in the United States reported professional reasons to be more important than non-professional ones for initial migratory intentions, and the latter to be more influential on their decision to return [41].

Among the reasons for staying behind, lack of resources was the second most common. Pakistan is classified under the lower middle income category by the World Bank, with a per capita income of USD1,120. This is far less than the basic requirement for the United States Residency Match, which requires finances in excess of USD20,000 (for licensing exams and clinical experience) [42]. Thus, the students who lack such resources are forced to stay back, although they may be willing to migrate if their financial deficiencies are overcome. A strange finding was that related to job satisfaction in Pakistan, which stood third in the list of reasons. However, other studies conducted in Pakistan also reported a higher than average score for job satisfaction in Pakistan, which is consistent with our data [7,23].

A large number of students had already started preparing for licensing exams and electives abroad, and an increasing trend was observed with increasing years in medical college. This can also be attributed to the easy availability of guidelines for preparation for exams/electives on the internet. As such, websites remained the most common sources of information in our sample. Most of these websites are run by the alumni of universities who were successful in pursuing their careers abroad. Therefore, healthy relations with the alumni can not only prove to be beneficial for the university in the long run, but may also play a definitive role in curbing migratory intentions in the future.

## Conclusion

A formidable proportion expressed migratory inclinations in our study. These intentions were facilitated by the pull factors of the recipient country such as lucrative salary, better quality of training and job satisfaction and the push factors related to Pakistan such as terrorism and harassment of doctors. The practices of respondents indicate that these migratory intentions may reach alarming rates in the future as reported by Talati, who estimated a shortfall of 58,000 to 451,000 physicians by the year 2020 [18].

### Limitations

The most important limitation for our study was that it was conducted in just one institute. Although, the college consists of a heterogeneous population coming from different backgrounds (as opposed to previous studies), it cannot predict the overall situation in the country. This implies that further studies should be conducted on a larger scale, with a more diverse set of institutes in order to minimize bias and for better generalization. Furthermore, convenient sampling was employed, which is not truly representative of the population under study. However, since this was just an observational study, the sampling method did seem to fulfill its purpose.

The low mean age of the respondents implies that our results cannot be considered useful for general policy making. However, specific policies targeting medical students will nevertheless prove beneficial in the long run, as the college life is the prime time for molding the attitudes of future physicians, and later interventions may receive a greater degree of disapproval. Thus, such strategies can serve as the first step in the formulation of general policies in the future.

### Recommendations

Serious steps need to be taken by the Government and concerned authorities in order to halt the continuous migration of physicians. If timely measures are not taken, disastrous consequences may follow, which may have a further detrimental effect on the already unstable healthcare system of the country. In view of the current situation, the authors would like to propose certain recommendations:

• Meetings should be held with the presidents of student societies to discuss the prevalent issues, and new policies should be drafted, which favor in-migration rather than out-migration. Since websites run by college alumni are an important factor in molding the way of thinking of the students, they should be contacted and discussions should be undertaken.

• The poor quality of training in Pakistan also formed one of the foremost factors favoring out migration. This warrants that the undergraduate and postgraduate training systems be revamped, with a more clinical approach rather than traditional rote learning.

• Special allocations (such as fringe benefits and contingent reward) should be made for those who do not intend to migrate in the future. For those who do, visa laws should be formulated that make it mandatory for them to serve their country for at least a certain time period ranging from 4-5 years. Individuals who have either completed, or are still pursuing their postgraduate education abroad should be encouraged to return.

• Special attention should be paid to ensure the security of health personnel. Unnecessary coercion and physical assaults should be strictly taken care of.

## Abbreviations

IMG: International Medical Graduate; USMLE: United States Medical Licensing Examination; PLAB: Professional and Linguistic Assessments Board Examination; MCCEE: Medical Council of Canada Evaluating Examination; AMC: Australian Medical Council Examination; MOH: Ministry of Health.

## Competing interests

The authors declare that they have no conflicts of interests. The authors did not receive any funding or remuneration for this work.

## Authors’ contributions

AS conceived the topic of the study. AS and AN were involved in designing the study and analyzing data. AS, AN, KS, SN and YB were involved in data collection. AS, AN, KS, SN and YB were involved in drafting the manuscript, listed in decreasing order of their contributions. All authors have read and approved the final manuscript.
